# Supplementary material for: Designing and immunomolecular analysis of a new broad-spectrum multiepitope vaccine against divergent human papillomavirus types
Source: PLoS One. 2024 Dec 2;19(12):e0311351. doi: 10.1371/journal.pone.0311351 (PMC11611089; doi:10.1371/journal.pone.0311351)
Supplement: S1 File — (DOCX) [file pone.0311351.s001.docx]

| Table S1. Representation of retrieved sequences along with their accession number in UniProt | | |
| --- | --- | --- |
| HPV type | **Accession number** | **Amino acid sequence** |
| 6 | P69899 | MWRPSDSTVYVPPPNPVSKVVATDAYVTRTNIFYHASSSRLLAVGHPYFSIKRANKTVVP  KVSGYQYRVFKVVLPDPNKFALPDSSLFDPTTQRLVWACTGLEVGRGQPLGVGVSGHPFL  NKYDDVENSGSGGNPGQDNRVNVGMDYKQTQLCMVGCAPPLGEHWGKGKQCTNTPVQAGD  CPPLELITSVIQDGDMVDTGFGAMNFADLQTNKSDVPIDICGTTCKYPDYLQMAADPYGD  RLFFFLRKEQMFARHFFNRAGEVGEPVPDTLIIKGSGNRTSVGSSIYVNTPSGSLVSSEA  QLFNKPYWLQKAQGHNNGICWGNQLFVTVVDTTRSTNMTLCASVTTSSTYTNSDYKEYMR  HVEEYDLQFIFQLCSITLSAEVMAYIHTMNPSVLEDWNFGLSPPPNGTLEDTYRYVQSQA  ITCQKPTPEKEKPDPYKNLSFWEVNLKEKFSSELDQYPLGRKFLLQSGYRGRSSIRTGVK  RPAVSKASAAPKRKRAKTKR |
| 11 | P04012 | MWRPSDSTVYVPPPNPVSKVVATDAYVKRTNIFYHASSSRLLAVGHPYYSIKKVNKTVVP  KVSGYQYRVFKVVLPDPNKFALPDSSLFDPTTQRLVWACTGLEVGRGQPLGVGVSGHPLL  NKYDDVENSGGYGGNPGQDNRVNVGMDYKQTQLCMVGCAPPLGEHWGKGTQCSNTSVQNG  DCPPLELITSVIQDGDMVDTGFGAMNFADLQTNKSDVPLDICGTVCKYPDYLQMAADPYG  DRLFFYLRKEQMFARHFFNRAGTVGEPVPDDLLVKGGNNRSSVASSIYVHTPSGSLVSSE  AQLFNKPYWLQKAQGHNNGICWGNHLFVTVVDTTRSTNMTLCASVSKSATYTNSDYKEYM  RHVEEFDLQFIFQLCSITLSAEVMAYIHTMNPSVLEDWNFGLSPPPNGTLEDTYRYVQSQ  AITCQKPTPEKEKQDPYKDMSFWEVNLKEKFSSELDQFPLGRKFLLQSGYRGRTSARTGI  KRPAVSKPSTAPKRKRTKTKK |
| 42 | I4GPX8 | MSVWRPSDNKVYLPPPPVSKVVSTDEYVQRTNYFYHASSSRLLVVGHPYYSITKRPNKTS  IPKVSGLQYRVFRVRLPDPNKFTLPETNLYNPDTQRMVWACVGLEVGRGQPLGVGISGHP  LLNKLDDTENAPTYGGGPGTDNRENVSMDYKQTQLCLVGCKPAIGEHWGKGTACTPQSNG  DCPPLELKNSVIQDGDMVDVGFGALDFGALQSSKAEVPLDIVNSITKYPDYLKMSAEAYG  DSMFFFLRREQMFVRHLFNRAGAIGEPVPDELYTKAANNASGRHNLGSSIYYPTPSGSMV  TSDAQLFNKPYWLQQAQGHNNGICWGDQLFLTVVDTTRSTNMTLCATATSGDTYTAANFK  EYLRHAEEYDVQFIFQLCKITLTVEVMSYIHNMNPNILEEWNVGVAPPPSGTLEDSYRYV  QSEAIRCQAKVTTPEKKDPYSDFSFWEVNLSEKFSTDLDQFPLGRKFLLQAGLRARPKLS  VGKRKASTAKSVSSAKRKKTHK |
| 43 | P50815 | MALIFICILHCGIWANAVNAFHIFLQITMWRLNDNKVYLPPPGPIASIVSTDEYVQRTNL  FYYAGSSRLLAVGHPYFPLKNSSGKITVPKVSGYQYRVFRVKLPDPNKFGFSETTLVTSD  TQRLVWGCVGVEIGRGQPLGVGISGHPYLNKYDDTENPSGYGTSPGQDNRENVAMDYKQT  QLCIVGCTPPMGEYWGQGVPCNASGVTQGDCPVIELKSEVIQDGDMVDTGFGAMDFASLQ  ASKSDVPLDLVNTKSKYPDYLGMAAEPYGNSLFFFLRREQMFLRHFFNKAGKTGDVVPSD  MYIAGSNTRSKIADSIYFSTPSGSLVTSDSQLFNKPLWIQKAQGHNNGICFGNQLFVTVV  DTTRSTNLTLCASTDPTVPSTYDNAKFKEYLRHVEEYDLQFIFQLCIITLNPEVMTYIHT  MDPTLLEDWNFGVSPPASASLEDTYRFLSNKAIACQKNAPPKEREDPYKKYTFWDINLTE  KFSAQLTQFPLGRKFVMQAGLRPKPKLKTVKRSAPSSSTSAPASKRKKTKR |
| 44 | P50816 | MWRPSENQVYVPPPAPVSKVIPTDAYVKRTNIYYHASSSRLLAVGNPYFAIRPANKTLVP  KVSGFQYRVFKMVLPDPNKFALPDTSIYDPTTQRLVWACIGLEVGRGQPLGVGISGHPLL  NKLDDVENSASYAAGPGQDNRVNVAMDYKQTQLCLVGCAPPLGEHWGKGKQCNNVSVKDG  DCPPLELITSVIEDGDMVDTGFGAMNFAELQPNKSDVPLDICTATCKYPDYLQMAADPYG  DRLFFYLRKEQMFARHFFNRAGTVGEDVSQDLVIKSASKNTVPNAIYFNTPSGSLVSSET  QLFNKPFWLQKAQGHNNGICWGNQLFVTVVDTTRSTNMTICAATTQSPPSTYTSEQYKQY  MRHVEEFDLQFMFQLCSITLTAEVMAYLHTMNAGILEQWNFGLSPPPNGTLEDKYRYVQS  QAITCQKPPPEKAKQDPYAKLSFWEVDLREKFSSELDQYPLGRKFLLQTGVQARSSVRVG  RKRPASAATSSSKQKRSRKK |
| 16 | P03101 | MSLWLPSEATVYLPPVPVSKVVSTDEYVARTNIYYHAGTSRLLAVGHPYFPIKKPNNNKI  LVPKVSGLQYRVFRIHLPDPNKFGFPDTSFYNPDTQRLVWACVGVEVGRGQPLGVGISGH  PLLNKLDDTENASAYAANAGVDNRECISMDYKQTQLCLIGCKPPIGEHWGKGSPCTNVAV  NPGDCPPLELINTVIQDGDMVDTGFGAMDFTTLQANKSEVPLDICTSICKYPDYIKMVSE  PYGDSLFFYLRREQMFVRHLFNRAGAVGENVPDDLYIKGSGSTANLASSNYFPTPSGSMV  TSDAQIFNKPYWLQRAQGHNNGICWGNQLFVTVVDTTRSTNMSLCAAISTSETTYKNTNF  KEYLRHGEEYDLQFIFQLCKITLTADVMTYIHSMNSTILEDWNFGLQPPPGGTLEDTYRF  VTSQAIACQKHTPPAPKEDPLKKYTFWEVNLKEKFSADLDQFPLGRKFLLQAGLKAKPKF  TLGKRKATPTTSSTSTTAKRKKRKL |
| 18 | P06794 | MCLYTRVLILHYHLLPLYGPLYHPRPLPLHSILVYMVHIIICGHYIILFLRNVNVFPIFL  QMALWRPSDNTVYLPPPSVARVVNTDDYVTPTSIFYHAGSSRLLTVGNPYFRVPAGGGNK  QDIPKVSAYQYRVFRVQLPDPNKFGLPDTSIYNPETQRLVWACAGVEIGRGQPLGVGLSG  HPFYNKLDDTESSHAATSNVSEDVRDNVSVDYKQTQLCILGCAPAIGEHWAKGTACKSRP  LSQGDCPPLELKNTVLEDGDMVDTGYGAMDFSTLQDTKCEVPLDICQSICKYPDYLQMSA  DPYGDSMFFCLRREQLFARHFWNRAGTMGDTVPQSLYIKGTGMPASPGSCVYSPSPSGSI  VTSDSQLFNKPYWLHKAQGHNNGVCWHNQLFVTVVDTTPSTNLTICASTQSPVPGQYDAT  KFKQYSRHVEEYDLQFIFQLCTITLTADVMSYIHSMNSSILEDWNFGVPPPPTTSLVDTY  RFVQSVAITCQKDAAPAENKDPYDKLKFWNVDLKEKFSLDLDQYPLGRKFLVQAGLRRKP  TIGPRKRSAPSATTSSKPAKRVRVRARK |
| 31 | P17388 | MSLWRPSEATVYLPPVPVSKVVSTDEYVTRTNIYYHAGSARLLTVGHPYYSIPKSDNPKK  IVVPKVSGLQYRVFRVRLPDPNKFGFPDTSFYNPETQRLVWACVGLEVGRGQPLGVGISG  HPLLNKFDDTENSNRYAGGPGTDNRECISMDYKQTQLCLLGCKPPIGEHWGKGSPCSNNA  ITPGDCPPLELKNSVIQDGDMVDTGFGAMDFTALQDTKSNVPLDICNSICKYPDYLKMVA  EPYGDTLFFYLRREQMFVRHFFNRSGTVGESVPTDLYIKGSGSTATLANSTYFPTPSGSM  VTSDAQIFNKPYWMQRAQGHNNGICWGNQLFVTVVDTTRSTNMSVCAAIANSDTTFKSSN  FKEYLRHGEEFDLQFIFQLCKITLSADIMTYIHSMNPAILEDWNFGLTTPPSGSLEDTYR  FVTSQAITCQKTAPQKPKEDPFKDYVFWEVNLKEKFSADLDQFPLGRKFLLQAGYRARPK  FKAGKRSAPSASTTTPAKRKKTKK |
| 33 | P06416 | MSVWRPSEATVYLPPVPVSKVVSTDEYVSRTSIYYYAGSSRLLAVGHPYFSIKNPTNAKK  LLVPKVSGLQYRVFRVRLPDPNKFGFPDTSFYNPDTQRLVWACVGLEIGRGQPLGVGISG  HPLLNKFDDTETGNKYPGQPGADNRECLSMDYKQTQLCLLGCKPPTGEHWGKGVACTNAA  PANDCPPLELINTIIEDGDMVDTGFGCMDFKTLQANKSDVPIDICGSTCKYPDYLKMTSE  PYGDSLFFFLRREQMFVRHFFNRAGTLGEAVPDDLYIKGSGTTASIQSSAFFPTPSGSMV  TSESQLFNKPYWLQRAQGHNNGICWGNQVFVTVVDTTRSTNMTLCTQVTSDSTYKNENFK  EYIRHVEEYDLQFVFQLCKVTLTAEVMTYIHAMNPDILEDWQFGLTPPPSASLQDTYRFV  TSQAITCQKTVPPKEKEDPLGKYTFWEVDLKEKFSADLDQFPLGRKFLLQAGLKAKPKLK  RAAPTSTRTSSAKRKKVKK |
| 35 | P27232 | MSLWRSNEATVYLPPVSVSKVVSTDEYVTRTNIYYHAGSSRLLAVGHPYYAIKKQDSNKI  AVPKVSGLQYRVFRVKLPDPNKFGFPDTSFYDPASQRLVWACTGVEVGRGQPLGVGISGH  PLLNKLDDTENSNKYVGNSGTDNRECISMDYKQTQLCLIGCRPPIGEHWGKGTPCNANQV  KAGECPPLELLNTVLQDGDMVDTGFGAMDFTTLQANKSDVPLDICSSICKYPDYLKMVSE  PYGDMLFFYLRREQMFVRHLFNRAGTVGETVPADLYIKGTTGTLPSTSYFPTPSGSMVTS  DAQIFNKPYWLQRAQGHNNGICWSNQLFVTVVDTTRSTNMSVCSAVSSSDSTYKNDNFKE  YLRHGEEYDLQFIFQLCKITLTADVMTYIHSMNPSILEDWNFGLTPPPSGTLEDTYRYVT  SQAVTCQKPSAPKPKDDPLKNYTFWEVDLKEKFSADLDQFPLGRKFLLQAGLKARPNFRL  GKRAAPASTSKKSSTKRRKVKS |
| 39 | P24838 | MAMWRSSDSMVYLPPPSVAKVVNTDDYVTRTGIYYYAGSSRLLTVGHPYFKVGMNGGRKQ  DIPKVSAYQYRVFRVTLPDPNKFSIPDASLYNPETQRLVWACVGVEVGRGQPLGVGISGH  PLYNRQDDTENSPFSSTTNKDSRDNVSVDYKQTQLCIIGCVPAIGEHWGKGKACKPNNVS  TGDCPPLELVNTPIEDGDMIDTGYGAMDFGALQETKSEVPLDICQSICKYPDYLQMSADV  YGDSMFFCLRREQLFARHFWNRGGMVGDAIPAQLYIKGTDIRANPGSSVYCPSPSGSMVT  SDSQLFNKPYWLHKAQGHNNGICWHNQLFLTVVDTTRSTNFTLSTSIESSIPSTYDPSKF  KEYTRHVEEYDLQFIFQLCTVTLTTDVMSYIHTMNSSILDNWNFAVAPPPSASLVDTYRY  LQSAAITCQKDAPAPEKKDPYDGLKFWNVDLREKFSLELDQFPLGRKFLLQARVRRRPTI  GPRKRPAASTSSSSATKHKRKRVSK |
| 45 | P36741 | MAHNIIYGHGIIIFLKNVNVFPIFLQMALWRPSDSTVYLPPPSVARVVSTDDYVSRTSIF  YHAGSSRLLTVGNPYFRVVPNGAGNKQAVPKVSAYQYRVFRVALPDPNKFGLPDSTIYNP  ETQRLVWACVGMEIGRGQPLGIGLSGHPFYNKLDDTESAHAATAVITQDVRDNVSVDYKQ  TQLCILGCVPAIGEHWAKGTLCKPAQLQPGDCPPLELKNTIIEDGDMVDTGYGAMDFSTL  QDTKCEVPLDICQSICKYPDYLQMSADPYGDSMFFCLRREQLFARHFWNRAGVMGDTVPT  DLYIKGTSANMRETPGSCVYSPSPSGSIITSDSQLFNKPYWLHKAQGHNNGICWHNQLFV  TVVDTTRSTNLTLCASTQNPVPSTYDPTKFKQYSRHVEEYDLQFIFQLCTITLTAEVMSY  IHSMNSSILENWNFGVPPPPTTSLVDTYRFVQSVAVTCQKDTTPPEKQDPYDKLKFWTVD  LKEKFSSDLDQYPLGRKFLVQAGLRRRPTIGPRKRPAASTSTASTASRPAKRVRIRSKK |
| 51 | P26536 | MALWRTNDSKVYLPPAPVSRIVNTEEYITRTGIYYYAGSSRLITLGHPYFPIPKTSTRAA  IPKVSAFQYRVFRVQLPDPNKFGLPDPNLYNPDTDRLVWGCVGVEVGRGQPLGVGLSGHP  LFNKYDDTENSRIANGNAQQDVRDNTSVDNKQTQLCIIGCAPPIGEHWGIGTTCKNTPVP  PGDCPPLELVSSVIQDGDMIDTGFGAMDFAALQATKSDVPLDISQSVCKYPDYLKMSADT  YGNSMFFHLRREQIFARHYYNKLVGVGEDIPNDYYIKGSGNGRDPIESYIYSATPSGSMI  TSDSQIFNKPYWLHRAQGHNNGICWNNQLFITCVDTTRSTNLTISTATAAVSPTFTPSNF  KQYIRHGEEYELQFIFQLCKITLTTEVMAYLHTMDPTILEQWNFGLTLPPSASLEDAYRF  VRNAATSCQKDTPPQAKPDPLAKYKFWDVDLKERFSLDLDQFALGRKFLLQVGVQRKPRP  GLKRPASSASSSSSSSAKRKRVKK |
| 52 | Q05138 | MVQILFYILVIFYYVAGVNVFHIFLQMSVWRPSEATVYLPPVPVSKVVSTDEYVSRTSIY  YYAGSSRLLTVGHPYFSIKNTSSGNGKKVLVPKVSGLQYRVFRIKLPDPNKFGFPDTSFY  NPETQRLVWACTGLEIGRGQPLGVGISGHPLLNKFDDTETSNKYAGKPGIDNRECLSMDY  KQTQLCILGCKPPIGEHWGKGTPCNNNSGNPGDCPPLQLINSVIQDGDMVDTGFGCMDFN  TLQASKSDVPIDICSSVCKYPDYLQMASEPYGDSLFFFLRREQMFVRHFFNRAGTLGDPV  PGDLYIQGSNSGNTATVQSSAFFPTPSGSMVTSESQLFNKPYWLQRAQGHNNGICWGNQL  FVTVVDTTRSTNMTLCAEVKKESTYKNENFKEYLRHGEEFDLQFIFQLCKITLTADVMTY  IHKMDATILEDWQFGLTPPPSASLEDTYRFVTSTAITCQKNTPPKGKEDPLKDYMFWEVD  LKEKFSADLDQFPLGRKFLLQAGLQARPKLKRPASSAPRTSTKKKKVKR |
| 56 | P36743 | MMLPMMYIYRDPPLHYGLCIFLDVGAVNVFPIFLQMATWRPSENKVYLPPTPVSKVVATD  SYVKRTSIFYHAGSSRLLAVGHPYYSVTKDNTKTNIPKVSAYQYRVFRVRLPDPNKFGLP  DTNIYNPDQERLVWACVGLEVGRGQPLGAGLSGHPLFNRLDDTESSNLANNNVIEDSRDN  ISVDGKQTQLCIVGCTPAMGEHWTKGAVCKSTQVTTGDCPPLALINTPIEDGDMIDTGFG  AMDFKVLQESKAEVPLDIVQSTCKYPDYLKMSADAYGDSMWFYLRREQLFARHYFNRAGK  VGETIPAELYLKGSNGREPPPSSVYVATPSGSMITSEAQLFNKPYWLQRAQGHNNGICWG  NQLFVTVVDTTRSTNMTISTATEQLSKYDARKINQYLRHVEEYELQFVFQLCKITLSAEV  MAYLHNMNANLLEDWNIGLSPPVATSLEDKYRYVRSTAITCQREQPPTEKQDPLAKYKFW  DVNLQDSFSTDLDQFPLGRKFLMQLGTRSKPAVATSKKRSAPTSTSTPAKRKRR |
| 58 | P26535 | MVLILCCTLAILFCVADVNVFHIFLQMSVWRPSEATVYLPPVPVSKVVSTDEYVSRTSIY  YYAGSSRLLAVGNPYFSIKSPNNNKKVLVPKVSGLQYRVFRVRLPDPNKFGFPDTSFYNP  DTQRLVWACVGLEIGRGQPLGVGVSGHPYLNKFDDTETSNRYPAQPGSDNRECLSMDYKQ  TQLCLIGCKPPTGEHWGKGVACNNNAAATDCPPLELFNSIIEDGDMVDTGFGCMDFGTLQ  ANKSDVPIDICNSTCKYPDYLKMASEPYGDSLFFFLRREQMFVRHFFNRAGKLGEAVPDD  LYIKGSGNTAVIQSSAFFPTPSGSIVTSESQLFNKPYWLQRAQGHNNGICWGNQLFVTVV  DTTRSTNMTLCTEVTKEGTYKNDNFKEYVRHVEEYDLQFVFQLCKITLTAEIMTYIHTMD  SNILEDWQFGLTPPPSASLQDTYRFVTSQAITCQKTAPPKEKEDPLNKYTFWEVNLKEKF  SADLDQFPLGRKFLLQSGLKAKPRLKRSAPTTRAPSTKRKKVKK |
| 59 | Q81971 | MALWRSSDNKVYLPPPSVAKVVSTDEYVTRTSIFYHAGSSRLLTVGHPYFKVPKGGNGRQ  DVPKVSAYQYRVFRVKLPDPNKFGLPDNTVYDPNSQRLVWACVGVEIGRGQPLGVGLSGH  PLYNKLDDTENSHVASAVDTKDTRDNVSVDYKQTQLCIIGCVPAIGEHWTKGTACKPTTV  VQGDCPPLELINTPIEDGDMVDTGYGAMDFKLLQDNKSEVPLDICQSICKYPDYLQMSAD  AYGDSMFFCLRREQVFARHFWNRSGTMGDQLPESLYIKGTDIRANPGSYLYSPSPSGSVV  TSDSQLFNKPYWLHKAQGLNNGICWHNQLFLTVVDTTRSTNLSVCASTTSSIPNVYTPTS  FKEYARHVEEFDLQFIFQLCKITLTTEVMSYIHNMNTTILEDWNFGVTPPPTASLVDTYR  FVQSAAVTCQKDTAPPVKQDPYDKLKFWPVDLKERFSADLDQFPLGRKFLLQLGARPKPT  IGPRKRAAPAPTSTPSPKRVKRRKSSRK |

**Table S2. The predicted LBL epitopes of HPV L1 protein for multiepitope vaccine construction.**

| # | **Epitope/Start position** | **Length** | **Antigenicity score** | **Allergenicity** | **Toxicity** | **Conservancy**  **(≥70%)** | **Estimated half-life** | **GRAVY** |
| --- | --- | --- | --- | --- | --- | --- | --- | --- |
| 1 | NFGLSPPPNGTL  (HPV6-398) | 12 | 1.0253 | NO | NO | Type 6: 100%  Type 11: 100%  Type 44: 100%  Type 16: 83.3%  Type 35: 83.3% | 1.4 hours (mammalian reticulocytes, in vitro)  3 min (yeast, in vivo)  >10 hours (Escherichia coli, in vivo) | -0.308 |
| 2 | LLAVGHPYFSIKRANK  (HPV6-41) | 16 | 0.9748 | NO | NO | Type 6: 100%  Type 11: 81.2%  Type 16: 75%  Type 33: 75%  Type 44: 75%  Type 58: 75% | 5.5 hours (mammalian reticulocytes, in vitro)  3 min (yeast, in vivo)  2 min (Escherichia coli, in vivo) | -0.025 |
| 3 | VSGYQYRVFKVVLPD  (HPV6-62) | 15 | 0.9381 | NO | NO | Type 6: 100%  Type 11: 100%  Type 43: 86.7%  Type 44: 86.7%  Type 18: 80%  Type 31: 80%  Type 33: 80%  Type 35: 80%  Type 39: 80%  Type 42: 80%  Type 45: 80%  Type 56: 80%  Type 58: 80%  Type 59: 80%  Type 16: 73.3%  Type 51: 73.3%  Type 52: 73.3% | 100 hours (mammalian reticulocytes, in vitro)  >20 hours (yeast, in vivo)  >10 hours (Escherichia coli, in vivo) | 0.173 |
| 4 | TSSTYTNSDYKE  (HPV6-346) | 12 | 0.7409 | NO | NO | Type 6: 100%  Type 11: 83.3% | 7.2 hours (mammalian reticulocytes, in vitro)  >20 hours (yeast, in vivo)  >10 hours (Escherichia coli, in vivo) | -1.792 |
| 5 | PTPEKEKPDPYKNLSF  (HPV6-426) | 16 | 0.6523 | NO | NO | Type 6: 100%  Type 11: 81.2% | >20 hours (mammalian reticulocytes, in vitro)  >20 hours (yeast, in vivo)  ? (Escherichia coli, in vivo) | -1.769 |
| 6 | NKYDDVENSGSGGNPGQDN  (HPV6-121) | 19 | 0.5395 | NO | NO | Type 6: 100% | 1.4 hours (mammalian reticulocytes, in vitro)  3 min (yeast, in vivo)  >10 hours (Escherichia coli, in vivo) | -1.963 |
| 7 | VSKVVATDAYVTRTNI  (HPV6-17) | 16 | 0.4357 | NO | NO | Type 6: 100%  Type 11: 93.7%  Type 31: 87.5%  Type 35: 87.5%  Type 16: 81.2%  Type 44: 81.2%  Type 56: 81.2%  Type 33: 75%  Type 39: 75%  Type 42: 75%  Type 52: 75%  Type 58: 75%  Type 59: 75% | 100 hours (mammalian reticulocytes, in vitro)  >20 hours (yeast, in vivo)  >10 hours (Escherichia coli, in vivo) | 0.331 |
| 8 | SSTYTNSDYKEYMRHVE  (HPV6-347) | 17 | 0.4118 | NO | NO | Type 6: 100%  Type 11: 94.1%  Type 44: 70.6% | 1.9 hours (mammalian reticulocytes, in vitro)  >20 hours (yeast, in vivo)  >10 hours (Escherichia coli, in vivo) | -1.600 |
| 9 | KRKATPTTSSTSTTAKRK  (HPV16-484) | 18 | 1.0324 | NO | NO | Type 16: 100% | 1.3 hours (mammalian reticulocytes, in vitro)  3 min (yeast, in vivo)  3 min (Escherichia coli, in vivo) | -1.622 |
| 10 | STSETTYKNT  (HPV16-349) | 10 | 0.6776 | NO | NO | Type 16: 100% | 1.9 hours (mammalian reticulocytes, in vitro)  >20 hours (yeast, in vivo)  >10 hours (Escherichia coli, in vivo) | -1.660 |
| 11 | TVYLPPVPVSKVVS  (HPV16-10) | 14 | 0.6496 | NO | NO | Type 16: 100%  Type 31: 100%  Type 33: 100%  Type 52: 100%  Type 58:100%  Type 35: 92.9%  Type 42: 85.7%  Type 56: 78.6%  Type 45: 71.4%  Type 59: 71.4% | 7.2 hours (mammalian reticulocytes, in vitro)  >20 hours (yeast, in vivo)  >10 hours (Escherichia coli, in vivo) | 0.893 |
| 12 | EATVYLPPVPVSKVVS  (HPV16-8) | 16 | 0.5634 | NO | NO | Type 16: 100%  Type 31: 100%  Type 33: 100%  Type 52: 100%  Type 58:100%  Type 35: 93.7%  Type 42: 75.7%  Type 56: 75% | 1 hours (mammalian reticulocytes, in vitro)  30 min (yeast, in vivo)  >10 hours (Escherichia coli, in vivo) | 0.675 |
| 13 | SETTYKNTNFKEYLRHGEEYD  (HPV16-351) | 16 | 0.5617 | NO | NO | Type 16: 100%  Type 35: 85.7%  Type 52: 81%  Type 31: 76.2%  Type 33:76.2%  Type 58: 76.2%  Type 42: 71.4% | 1.9 hours (mammalian reticulocytes, in vitro)  >20 hours (yeast, in vivo)  >10 hours (Escherichia coli, in vivo) | -1.933 |
| 14 | EATVYLPPVPVSKVV  (HPV16-351) | 15 | 0.5549 | NO | NO | Type 16: 100%  Type 31: 100%  Type 33: 100%  Type 52: 100%  Type 58:100%  Type 35: 93.3%  Type 56: 80%  Type 42: 73.3% | 1 hours (mammalian reticulocytes, in vitro)  30 min (yeast, in vivo)  >10 hours (Escherichia coli, in vivo) | 0.773 |
| 15 | LLAVGHPYFPIKKPNN  (HPV16-42) | 16 | 0.5151 | NO | NO | Type 16: 100%  Type 33: 81.2%  Type 58: 81.2%  Type 6: 75%  Type 11: 75% | 5.5 hours (mammalian reticulocytes, in vitro)  3 min (yeast, in vivo)  2 min (Escherichia coli, in vivo) | -0.225 |
| 16 | PDPNKFGFPDTSFYNPDT  (HPV16-78) | 18 | 0.4558 | NO | NO | Type 16: 100%  Type 33: 100%  Type 58: 100%  Type 31: 94.4%  Type 52: 94.4%  Type 18: 83.3%  Type 35: 83.3%  Type 51: 77.8%  Type 56: 77.8%  Type 39: 72.2%  Type 42: 72.2%  Type 44: 72.2%  Type 5: 72.2% | >20 hours (mammalian reticulocytes, in vitro)  >20 hours (yeast, in vivo)  ? (Escherichia coli, in vivo) | -1.294 |
| 17 | KPTIGPRKRSAPSATT  (HPV18-539) | 16 | 1.5692 | NO | NO | Type 18: 100%  Type 59: 75% | 1.3 hours (mammalian reticulocytes, in vitro).  3 min (yeast, in vivo).  3 min (Escherichia coli, in vivo | -1.100 |
| 18 | GDMVDTGYGA  (HPV18-259) | 10 | 1.3932 | NO | NO | Type 18: 100%  Type 45: 100%  Type 59: 100%  Type 6: 90%  Type 11: 90%  Type 16: 90%  Type 31: 90%  Type 35: 90%  Type 39: 90%  Type 43: 90%  Type 44: 90%  Type 33: 80%  Type 42: 80%  Type 51: 80%  Type 52:80%  Type 56: 80%  Type 58: 80% | 30 hours (mammalian reticulocytes, in vitro).  >20 hours (yeast, in vivo).  >10 hours (Escherichia coli, in vivo) | -0.230 |
| 19 | RRKPTIGPRKRSAPSATTSSKPAKR  (HPV18-537) | 25 | 1.4412 | NO | NO | Type 18: 100% | 1 hours (mammalian reticulocytes, in vitro).  2 min (yeast, in vivo).  2 min (Escherichia coli, in vivo) | -1.612 |
| 20 | NFGVPPPPTTSLVD  (HPV18-465) | 14 | 0.7282 | NO | NO | Type 18: 100%  Type 45: 100%  Type 59: 85.7%  Type 39: 71.4% | 1.4 hours (mammalian reticulocytes, in vitro).  3 min (yeast, in vivo).  >10 hours (Escherichia coli, in vivo) | -0.071 |
| 21 | KAQGHNNGVCWHNQ  (HPV18-376) | 14 | 0.6462 | NO | NO | Type 18: 100%  Type 39: 92.9%  Type 45: 92.9%  Type 6: 85.7%  Type 44: 85.7%  Type 59: 85.7%  Type 11: 78.6%  Type 16: 78.6%  Type 31: 78.6%  Type 33: 78.6%  Type 35: 78.6%  Type 43: 78.6%  Type 51: 78.6%  Type 52: 78.6%  Type 56: 78.6%  Type 58: 78.6%  Type 42: 71.4% | 1.3 hours (mammalian reticulocytes, in vitro)  3 min (yeast, in vivo)  3 min (Escherichia coli, in vivo) | -1.500 |
| 22 | GVGLSGHPFYNKLDDT  (HPV18-175) | 16 | 0.5963 | NO | NO | Type 18: 100%  Type 45: 93.7%  Type 59: 93.7%  Type 16: 81.2%  Type 35: 81.2%  Type 42: 81.2%  Type 51: 81.2%  Type 6: 75%  Type 31: 75%  Type 33: 75%  Type 35: 75%  Type 39: 75%  Type 43: 75%  Type 44: 75%  Type 52: 75%  Type 56: 75%  Type 58: 75% | 30 hours (mammalian reticulocytes, in vitro).  >20 hours (yeast, in vivo).  >10 hours (Escherichia coli, in vivo). | -0.537 |
| 23 | PFYNKLDDTES  (HPV18-182) | 11 | 0.5200 | NO | NO | Type 18: 100%  Type 45: 100%  Type 59: 81.8%  Type 16: 72.7%  Type 35: 72.7%  Type 42: 72.7%  Type 56: 72.7% | >20 hours (mammalian reticulocytes, in vitro).  >20 hours (yeast, in vivo).  ? (Escherichia coli, in vivo) | -1.427 |
| 24 | YNKLDDTESSHAATSNVSEDVRDNVSVDY  (HPV18-184) | 29 | 0.5163 | NO | NO | Type 18: 100%  Type 45: 79.3% | 2.8 hours (mammalian reticulocytes, in vitro).  10 min (yeast, in vivo).  2 min (Escherichia coli, in vivo). | -1.048 |
| 25 | GHPFYNKLDDTESS  (HPV18-180) | 14 | 0.4739 | NO | NO | Type 18: 100%  Type 45: 92.7%  Type 59: 85.7%  Type 56: 78.6%  Type 6: 71.4%  Type 11: 71.4%  Type 16: 71.4%  Type 31: 71.4%  Type 39: 71.4%  Type 42: 71.4%  Type 44: 71.4%  Type 51: 71.4%  Type 52: 71.4%  Type 58: 71.4% | 30 hours (mammalian reticulocytes, in vitro)  >20 hours (yeast, in vivo)  >10 hours (Escherichia coli, in vivo) | -1.436 |

**Table S3. The predicted CTL epitopes of HPV L1 protein for multiepitope vaccine construction.**

| **#** | **Epitope** | **Length** | **Alleles** | **Antigenicity score** | **Immunogenicity score** | **Allergenicity** | **Toxicity** | **Conservancy**  **(≥70%)** | **Estimated half-life** | **GRAVY** |
| --- | --- | --- | --- | --- | --- | --- | --- | --- | --- | --- |
| 1 | DTGFGAMNF  (HPV6-198) | 9 | HLA-A*26:01, HLA-B*53:01, HLA-B*35:01, HLA-A*01:01, HLA-B*51:01, HLA-A*68:02, HLA-B*57:01, HLA-A*68:01, HLA-A*30:02, HLA-A*24:02, HLA-A*23:01, HLA-B*58:01, HLA-B*15:01, HLA-A*32:01, HLA-B*08:01, HLA-A*33:01, HLA-B*44:02, HLA-A*30:01, HLA-B*44:03, HLA-B*07:02, HLA-A*02:06, HLA-A*11:01, HLA-A*03:01, HLA-A*31:01, HLA-A*02:01, HLA-B*40:01, HLA-A*02:03, HLA-A*24:02, HLA-A*23:01 | 2.1472 | 0.0466 | NO | NO | Type 6: 100%  Type 11: 100%  Type 44: 100%  Type 43: 88.9%  Type 16: 88.9%  Type 31: 88.9%  Type 35: 88.9%  Type 51: 88.9%  Type 56: 88.9%  Type 18: 77.8%  Type 35: 77.8%  Type 39: 77.8%  Type 45: 77.8%  Type 52: 77.8%  Type 58: 77.8%  Type 59: 77.8% | 1.1 hours (mammalian reticulocytes, in vitro)  3 min (yeast, in vivo)  >10 hours (Escherichia coli, in vivo) | 0.089 |
| 2 | DTGFGAMNFA  (HPV6-198) | 10 | HLA-A*68:02, HLA-A*68:01, HLA-A*26:01, HLA-B*51:01, HLA-A*33:01, HLA-A*02:06, HLA-A*01:01, HLA-A*30:01, HLA-A*02:03,  HLA-A*02:01, HLA-B*57:01,  HLA-A*30:02, HLA-B*08:01, HLA-B*35:01, HLA-B*53:01, HLA-B*58:01, HLA-A*03:01, HLA-A*11:01, HLA-A*31:01, HLA-B*07:02, HLA-B*44:02, HLA-B*15:01, HLA-A*32:01, HLA-B*44:03, HLA-A*24:02, HLA-A*23:01, HLA-B*40:01 | 1.8988 | 0.0975 | NO | NO | Type 6: 100%  Type 11: 100%  Type 44: 100%  Type 43: 90%  Type 51: 90%  Type 16: 80%  Type 31: 80%  Type 35: 80%  Type 56: 80%  Type 18: 70%  Type 33: 70%  Type 39: 70%  Type 45: 70%  Type 52: 70%  Type 58: 70%  Type 59: 70% | 1.1 hours (mammalian reticulocytes, in vitro)  3 min (yeast, in vivo)  >10 hours (Escherichia coli, in vivo) | 0.260 |
| 3 | MNFADLQTNK  (HPV6-204) | 10 | HLA-A*68:01, HLA-A*11:01, HLA-A*03:01, HLA-A*33:01, HLA-A*31:01, HLA-A*30:01, HLA-A*30:02, HLA-B*57:01, HLA-B*58:01, HLA-A*01:01, HLA-A*26:01, HLA-A*68:02, HLA-A*24:02, HLA-A*23:01, HLA-A*32:01, HLA-B*51:01, HLA-B*15:01, HLA-A*02:06, HLA-B*35:01, HLA-B*44:02, HLA-A*02:01, HLA-A*02:03, HLA-B*08:01, HLA-B*44:03, HLA-B*53:01, HLA-B*07:02, HLA-B*40:01, | 1.1124 | 0.0081 | NO | NO | Type 6: 100%  Type 11: 100%  Type 44: 80% | 30 hours (mammalian reticulocytes, in vitro).  >20 hours (yeast, in vivo).  >10 hours (Escherichia coli, in vivo). | -0.830 |
| 4 | RLLAVGHPY  (HPV16-40) | 9 |  | 0.7359 | 0.12869 | NO | NO | Type 6: 100%  Type 11: 100%  Type 16: 100%  Type 33: 100%  Type 35: 100%  Type 43: 100%  Type 56: 100%  Type 31: 88.9%  Type 39: 88.9%  Type 42: 88.9%  Type 44: 88.9%  Type 52: 88.9%  Type 58: 88.9%  Type 59: 88.9%  Type 18: 77.8%  Type 45: 77.8% | 1 hours (mammalian reticulocytes, in vitro)  2 min (yeast, in vivo)  2 min (Escherichia coli, in vivo) | 0.289 |
| 5 | SRLLAVGHPY  (HPV16-39) | 10 |  | 0.5755 | 0.11626 | NO | NO | Type 6: 100%  Type 11: 100%  Type 16: 100%  Type 33: 100%  Type 35: 100%  Type 43: 100%  Type 56: 100%  Type 39: 90%  Type 42: 90%  Type 44: 90%  Type 52: 90%  Type 58: 90%  Type 59: 90%  Type 18: 80%  Type 31: 80%  Type 45: 80% | 1.9 hours (mammalian reticulocytes, in vitro)  >20 hours (yeast, in vivo)  >10 hours (Escherichia coli, in vivo) | 0.180 |
| 6 | KFGFPDTSFY  (HPV16-82) | 10 | HLA-A*30:02, HLA-B*58:01, HLA-B*57:01, HLA-B*15:01, HLA-A*03:01, HLA-A*01:01, HLA-A*30:01, HLA-A*32:01, HLA-A*31:01, HLA-A*11:01, HLA-A*23:01, HLA-B*35:01, HLA-A*24:02, HLA-A*26:01, HLA-A*68:01, HLA-B*53:01, HLA-B*44:03, HLA-A*33:01, HLA-B*44:02, HLA-B*51:01, HLA-A*02:06, HLA-A*02:01, HLA-B*08:01, HLA-B*07:02, HLA-A*02:03, HLA-B*40:01, HLA-A*68:02 | 1.1960 | 0.1049 | NO | NO | Type 16: 100%  Type 31: 100%  Type 33: 100%  Type 35: 100%  Type 52: 100%  Type 58: 100%  Type 18: 80%  Type 44: 70%  Type 56: 70% | 1.3 hours (mammalian reticulocytes, in vitro).  3 min (yeast, in vivo).  3 min (Escherichia coli, in vivo) | -0.380 |
| 7 | EATVYLPPV  (HPV16-8) | 9 | HLA-B*51:01, HLA-A*68:02, HLA-A*02:06, HLA-B*08:01, HLA-A*26:01, HLA-A*02:01, HLA-A*02:03, HLA-B*35:01, HLA-B*53:01, HLA-A*33:01, HLA-A*68:01, HLA-B*07:02, HLA-B*58:01, HLA-A*01:01, HLA-A*30:01, HLA-B*57:01, HLA-A*30:02, HLA-A*32:01, HLA-A*31:01, HLA-B*44:02, HLA-B*44:03, HLA-A*24:02, HLA-B*15:01, HLA-A*23:01, HLA-A*03:01, HLA-B*40:01, HLA-A*11:01 | 0.4206 | 0.0242 | NO | NO | Type 16: 100%  Type 31: 100%  Type 33: 100%  Type 35: 100%  Type 52: 100%  Type 58: 100% | 1 hours (mammalian reticulocytes, in vitro).  30 min (yeast, in vivo).  >10 hours (Escherichia coli, in vivo). | 0.589 |
| 8 | HVEEYDLQF  (HPV18-428) | 9 | HLA-B*35:01,  HLA-A*01:01,  HLA-B*53:01,  HLA-A*26:01,  HLA-B*15:01,  HLA-B*58:01,  HLA-A*32:01,  HLA-B*57:01,  HLA-A*02:06,  HLA-A*30:02,  HLA-B*08:01,  HLA-A*23:01,  HLA-A*24:02,  HLA-A*68:02,  HLA-B*51:01,  HLA-A*02:01,  HLA-A*68:01,  HLA-B*44:02,  HLA-B*07:02,  HLA-B*44:03,  HLA-B*40:01,  HLA-A*11:01,  HLA-A*02:03,  HLA-A*31:01,  HLA-A*30:01,  HLA-A*03:01, HLA-A*33:01 | 1.4499 | 0.07349 | NO | NO | Type 6: 100%  Type 18: 100%  Type 33: 100%  Type 39: 100%  Type 43: 100%  Type 45: 100%  Type 58: 100%  Type 11: 89.9%  Type 16: 89.9%  Type 35: 89.9%  Type 44: 89.9%  Type 56: 89.9%  Type 59: 89.9%  Type 31: 77.8%  Type 42: 77.8%  Type 51: 77.8%  Type 52: 77.8% | 3.5 hours (mammalian reticulocytes, in vitro).  10 min (yeast, in vivo)  >10 hours (Escherichia coli, in vivo) | -0.856 |
| 9 | LTVGNPYFR  (HPV18-104) | 9 | HLA-A*68:01, HLA-A*31:01, HLA-A*33:01, HLA-A*11:01, HLA-A*03:01, HLA-A*30:01, HLA-A*26:01, HLA-A*01:01, HLA-A*30:02, HLA-A*68:02, HLA-B*57:01, HLA-A*02:06, HLA-A*32:01, HLA-B*58:01, HLA-A*02:01, HLA-B*35:01, HLA-B*51:01, HLA-B*53:01, HLA-A*02:03, HLA-B*15:01, HLA-A*23:01, HLA-A*24:02, HLA-B*08:01, HLA-B*07:02, HLA-B*44:03, HLA-B*44:02, HLA-B*40:01 | 1.2652 | 0.0960 | NO | NO | Type 18: 100%  Type 45: 100%  Type 39: 77.8%  Type 44: 77.8%  Type 52: 77.8%  Type 58: 77.8%  Type 59: 77.8% | 5.5 hours (mammalian reticulocytes, in vitro).  3 min (yeast, in vivo).  2 min (Escherichia coli, in vivo). | -0.133 |
| 10 | LTVGNPYFRV  (HPV18-104) | 10 | HLA-A*68:02, HLA-A*68:01, HLA-A*02:06, HLA-B*57:01, HLA-A*02:03, HLA-A*31:01, HLA-A*02:01, HLA-B*58:01, HLA-A*33:01, HLA-A*11:01, HLA-A*30:01, HLA-A*01:01, HLA-B*51:01, HLA-A*26:01, HLA-A*30:02, HLA-A*03:01, HLA-A*32:01, HLA-A*23:01, HLA-A*24:02, HLA-B*08:01, HLA-B*15:01, HLA-B*53:01, HLA-B*07:02, HLA-B*40:01, HLA-B*35:01, HLA-B*44:02, HLA-B*44:03 | 1.0974 | 0.1559 | NO | NO | Type 18: 100%  Type 45: 100%  Type 39: 80%  Type 59: 80%  Type 44: 70%  Type 52: 70%  Type 58: 70% | 5.5 hours (mammalian reticulocytes, in vitro).  3 min (yeast, in vivo).  2 min (Escherichia coli, in vivo) | 0.300 |
| 11 | LLTVGNPYFR  (HPV18-103) | 10 | HLA-A*68:01, HLA-A*31:01, HLA-A*33:01, HLA-A*11:01, HLA-A*03:01, HLA-A*30:01, HLA-A*26:01, HLA-A*01:01, HLA-A*30:02, HLA-B*57:01, HLA-A*02:06, HLA-A*02:01, HLA-A*68:02, HLA-B*58:01, HLA-A*32:01, HLA-A*02:03, HLA-B*15:01, HLA-B*51:01, HLA-A*24:02, HLA-B*08:01, HLA-A*23:01, HLA-B*53:01, HLA-B*35:01, HLA-B*07:02, HLA-B*44:02, HLA-B*44:03, HLA-B*40:01 | 0.9048 | 0.1356 | NO | NO | Type 18: 100%  Type 45: 100%  Type 33: 80%  Type 44: 80%  Type 52: 80%  Type 58: 80%  Type 59: 80%  Type 6: 70%  Type 16: 70%  Type 31: 70%  Type 33: 70%  Type 43: 70% | 5.5 hours (mammalian reticulocytes, in vitro).  3 min (yeast, in vivo).  2 min (Escherichia coli, in vivo) | 0.260 |
| 12 | TVGNPYFRV  (HPV18-28) | 9 |  | **0.**8608 | 0.11925 | NO | NO | Type 18: 100%  Type 45: 100%  Type 39: 77.8%  Type 52: 77.8% | 7.2 hours (mammalian reticulocytes, in vitro)  >20 hours (yeast, in vivo)  >10 hours (Escherichia coli, in vivo) | -0.089 |
| 13 | NVFPIFLQMA  (HPV18-54) | 10 | HLA-A*68:02, HLA-A*02:06, HLA-A*02:03, HLA-A*02:01, HLA-A*68:01, HLA-A*26:01, HLA-A*30:01, HLA-A*33:01, HLA-B*51:01, HLA-A*30:02, HLA-B*57:01, HLA-A*31:01, HLA-B*08:01, HLA-A*03:01, HLA-A*32:01, HLA-B*35:01, HLA-A*11:01, HLA-A*01:01, HLA-B*58:01, HLA-B*15:01, HLA-B*53:01, HLA-B*07:02, HLA-A*24:02, HLA-A*23:01, HLA-B*40:01, HLA-B*44:03, HLA-B*44:02 | 0.8140 | 0.0596 | NO | NO | Type 18: 100%  Type 45: 100%  Type 56: 100%  Type 52: 80%  Type 58: 80% | 1.4 hours (mammalian reticulocytes, in vitro)  3 min (yeast, in vivo)  >10 hours (Escherichia coli, in vivo) | 1.320 |
| 14 | NVFPIFLQM  (HPV18-54) | 9 | HLA-A*68:02, HLA-A*26:01, HLA-A*02:06, HLA-B*35:01, HLA-A*68:01, HLA-A*32:01, HLA-B*51:01, HLA-B*57:01, HLA-A*02:01, HLA-B*53:01, HLA-A*33:01, HLA-B*15:01, HLA-A*30:02, HLA-B*08:01, HLA-A*02:03, HLA-B*58:01, HLA-A*30:01, HLA-A*23:01, HLA-A*11:01, HLA-A*31:01, HLA-A*24:02, HLA-B*07:02, HLA-A*03:01, HLA-A*01:01, HLA-B*44:03, HLA-B*40:01, HLA-B*44:02 | 0.8028 | 0.1896 | NO | NO | Type 18: 100%  Type 45: 100%  Type 56: 100%  Type 52: 88.9%  Type 58: 88.9% | 1.4 hours (mammalian reticulocytes, in vitro).  3 min (yeast, in vivo).  >10 hours (Escherichia coli, in vivo) | 1.267 |
| 15 | RLLTVGNPYF  (HPV18-102) | 10 | HLA-A*32:01, HLA-B*15:01, HLA-B*57:01, HLA-A*30:02, HLA-B*58:01, HLA-A*23:01, HLA-A*02:06, HLA-A*24:02, HLA-A*02:01, HLA-A*31:01, HLA-A*30:01, HLA-A*03:01, HLA-A*02:03, HLA-A*01:01, HLA-B*08:01, HLA-B*07:02, HLA-A*26:01, HLA-B*53:01, HLA-B*35:01, HLA-B*51:01, HLA-B*44:02, HLA-B*44:03, HLA-A*11:01, HLA-B*40:01, HLA-A*33:01, HLA-A*68:01, HLA-A*68:02 | 0.7004 | 0.0910 | NO | NO | Type 18: 100%  Type 45: 100%  Type 39: 90%  Type 44: 90%  Type 52: 90%  Type 58: 90%  Type 59: 90%  Type 6: 80%  Type 16: 80%  Type 31: 80%  Type 33: 80%  Type 43: 80%  Type 11: 70%  Type 35: 70%  Type 42: 70%  Type 51: 70%  Type 56: 70% | 1 hours (mammalian reticulocytes, in vitro).  2 min (yeast, in vivo).  2 min (Escherichia coli, in vivo) | 0.260 |
| 16 | LLTVGNPYF  (HPV18-103) | 9 | HLA-B*15:01, HLA-B*58:01, HLA-A*24:02, HLA-A*02:06, HLA-B*57:01, HLA-A*23:01, HLA-A*32:01, HLA-B*35:01, HLA-A*02:01, HLA-B*53:01, HLA-A*30:02, HLA-A*01:01, HLA-B*08:01, HLA-A*02:03, HLA-A*26:01, HLA-B*51:01, HLA-B*07:02, HLA-A*30:01, HLA-A*31:01, HLA-A*03:01, HLA-B*44:02, HLA-A*33:01, HLA-A*68:02, HLA-B*40:01, HLA-B*44:03, HLA-A*68:01, HLA-A*11:01 | 0.6926 | 0.0695 | NO | NO | Type 18: 100%  Type 45: 100%  Type 39: 88.9%  Type 44: 88.9%  Type 52: 88.9%  Type 58: 88.9%  Type 59: 88.9%  Type 6: 77.8%  Type 16: 77.8%  Type 31: 77.8%  Type 33: 77.8%  Type 43: 77.8% | 5.5 hours (mammalian reticulocytes, in vitro).  3 min (yeast, in vivo).  2 min (Escherichia coli, in vivo) | 0.789 |
| 17 | GLSGHPFYNK  (HPV18-177) | 10 | HLA-A*03:01, HLA-A*03:01, HLA-A*11:01, HLA-A*30:01, HLA-A*31:01, HLA-A*68:01, HLA-A*30:02, HLA-A*32:01, HLA-A*33:01, HLA-A*02:01, HLA-A*01:01, HLA-A*02:03, HLA-B*57:01, HLA-A*02:06, HLA-B*58:01, HLA-B*15:01, HLA-A*26:01, HLA-A*68:02, HLA-A*24:02, HLA-A*23:01, HLA-B*08:01, HLA-B*51:01, HLA-B*44:02, HLA-B*44:03, HLA-B*07:02, HLA-B*53:01, HLA-B*40:01, HLA-B*35:01 | 0.5524 | 0.1044 | NO | NO | Type 18: 100%  Type 45: 100%  Type 59: 90%  Type 6: 80%  Type 51: 80%  Type 11: 70%  Type 16: 70%  Type 31: 70%  Type 33: 70%  Type 35: 70%  Type 39: 70%  Type 42: 70%  Type 43: 70%  Type 44: 70%  Type 52: 70%  Type 56: 70%  Type 58: 70% | 30 hours (mammalian reticulocytes, in vitro).  >20 hours (yeast, in vivo).  >10 hours (Escherichia coli, in vivo) | -0.850 |
| 18 | NVNVFPIFL  (HPV18-52) | 9 | HLA-A*68:02, HLA-A*02:06, HLA-A*02:01, HLA-B*08:01, HLA-B*51:01, HLA-A*02:03, HLA-A*32:01, HLA-A*26:01, HLA-A*30:01, HLA-A*68:01, HLA-A*33:01, HLA-B*53:01, HLA-B*58:01, HLA-A*30:02, HLA-B*57:01, HLA-A*31:01, HLA-B*35:01, HLA-A*01:01, HLA-B*07:02, HLA-A*24:02, HLA-A*23:01, HLA-B*15:01, HLA-A*11:01, HLA-A*03:01, HLA-B*44:02, HLA-B*40:01, HLA-B*44:03 | 0.5018 | 0.3237 | NO | NO | Type 18: 100%  Type 45: 100%  Type 56: 88.9%  Type 52: 77.8%  Type 58: 77.8% | 1.4 hours (mammalian reticulocytes, in vitro).  3 min (yeast, in vivo).  >10 hours (Escherichia coli, in vivo) | 1.522 |
| 19 | TTSLVDTYR | 9 |  | **0.4094** | 0.02682 | NO | NO | Type 18: 100%  Type 45: 100%  Type 59: 88.9%  Type 39: 77.8% | 7.2 hours (mammalian reticulocytes, in vitro)  >20 hours (yeast, in vivo)  >10 hours (Escherichia coli, in vivo) | -0.467 |

**Table S4. The predicted HTL epitopes of HPV L1 protein for multiepitope vaccine construction.**

| **#** | **Epitope** | **Length** | **Top Alleles** | **Antigenicity score** | **IFN-g score** | **Allergenicity** | **Toxicity** | **Conservancy**  **(≥70%)** | **Estimated half-life** | **GRAVY** |
| --- | --- | --- | --- | --- | --- | --- | --- | --- | --- | --- |
| 1 | KFLLQSGYRGRSSIR  (HPV6-462) | 15 | HLA-DRB5*01:01, HLA-DRB1*03:01, HLA-DRB4*01:01, HLA-DRB3*02:02, HLA-DRB1*15:01, HLA-DRB1*07:01, HLA-DRB3*01:01 | 1.0609 | 0.2297 | No | No | Type 6: 100%  Type 11: 86.7% | 1.3 hours (mammalian reticulocytes, in vitro).  3 min (yeast, in vivo).  3 min (Escherichia coli, in vivo) | -0.700 |
| 2 | HPYFSIKRANKTVVP  (HPV6-46) | 15 | HLA-DRB5*01:01,  HLA-DRB3*02:02, HLA-DRB1*07:01, HLA-DRB1*03:01, HLA-DRB1*15:01, HLA-DRB3*01:01, HLA-DRB4*01:01 | 1.0342 | 0.1004 | No | No | Type 6: 100%  Type 11: 80% | 3.5 hours (mammalian reticulocytes, in vitro).  10 min (yeast, in vivo).  >10 hours (Escherichia coli, in vivo) | -0.500 |
| 3 | RKFLLQSGYRGRSSI  (HPV6-461) | 15 | HLA-DRB5*01:01, HLA-DRB3*02:02, HLA-DRB1*15:01, HLA-DRB4*01:01, HLA-DRB1*07:01, HLA-DRB3*01:01, HLA-DRB1*03:01 | 0.6668 | 0.2443 | No | No | Type 6: 100%  Type 11: 86.7% | 1 hours (mammalian reticulocytes, in vitro).  2 min (yeast, in vivo).  2 min (Escherichia coli, in vivo) | -0.700 |
| 4 | WGNQLFVTVVDTTRSTN  (HPV6-321) | 17 | HLA-DRB5*01:01, HLA-DRB4*01:01, HLA-DRB3*01:01, HLA-DRB3*02:02, HLA-DRB1*07:01, HLA-DRB1*15:01, HLA-DRB1*03:01 | 0.6751 | 0.0315 | No | No | Type 6: 100%  Type 31: 100%  Type 44: 100%  Type 16: 100%  Type 52: 100%  Type 56: 100%  Type 58: 100%  Type 11: 94.1%  Type 33: 94.1%  Type 35: 94.1%  Type 43: 94.1%  Type 45: 94.1%  Type 18: 88.2%  Type 42: 88.2%  Type 39: 88.1%  Type 59: 88.1%  Type 51: 82.3% | 2.8 hours (mammalian reticulocytes, in vitro).  3 min (yeast, in vivo).  2 min (Escherichia coli, in vivo) | -0.247 |
| 5 | LGRKFLLQSGYRGRS  (HPV6-459) | 15 | HLA-DRB5*01:01, HLA-DRB3*02:02, HLA-DRB1*15:01, HLA-DRB4*01:01, HLA-DRB1*07:01, HLA-DRB3*01:01, HLA-DRB1*03:01 | 0.7207 | 0.5211 | No | No | Type 6: 100%  Type 11: 93.3%  Type 31: 80%  Type 42: 73.3%  Type 44: 73.3% | 5.5 hours (mammalian reticulocytes, in vitro).  3 min (yeast, in vivo).  2 min (Escherichia coli, in vivo) | -0.720 |
| 6 | PSEATVYLPPVPVSK  (HPV16-6) | 15 | HLA-DRB1*07:01, HLA-DRB1*15:01, HLA-DRB5*01:01, HLA-DRB3*02:02, HLA-DRB3*01:01, HLA-DRB4*01:01, HLA-DRB1*03:01 | 0.5687 | 0.0812 | No | No | Type 16: 100%  Type 31:100%  Type 33: 100%  Type 52: 100%  Type 58:100%  Type 35: 80%  Type 56: 80%  Type 42: 73.3% | >20 hours (mammalian reticulocytes, in vitro).  >20 hours (yeast, in vivo).  ? (Escherichia coli, in vivo) | 0.053 |
| 7 | EATVYLPPVPVSKVV  (HPV16-8) | 15 | HLA-DRB5*01:01, HLA-DRB3*02:02, HLA-DRB1*07:01, HLA-DRB1*15:01, HLA-DRB4*01:01, HLA-DRB3*01:01, HLA-DRB1*03:01 | 0.5549 | 0.01 | No | No | Type 16: 100%  Type 31: 100%  Type 33: 100%  Type 52: 100%  Type 58:100%  Type 35: 93.3%  Type 56: 80%  Type 42: 73.3% | 1 hours (mammalian reticulocytes, in vitro).  30 min (yeast, in vivo).  >10 hours (Escherichia coli, in vivo) | 0.773 |
| 8 | HSMNSSILEDWNFGVPPP  (HPV18-454) | 18 | HLA-DRB3*01:01, HLA-DRB1*03:01, HLA-DRB1*15:01, HLA-DRB3*02:02, HLA-DRB1*07:01, HLA-DRB4*01:01, HLA-DRB5*01:01 | 0.6607 | 0.1183 | No | No | Type 18: 100%  Type 45: 94.4%  Type 16: 83.3%  Type 35: 83.3%  Type 59: 77.8%  Type 6: 72.2%  Type 11: 72.2%  Type 31: 72.2%  Type 39: 72.2% | 3.5 hours (mammalian reticulocytes, in vitro).  10 min (yeast, in vivo).  >10 hours (Escherichia coli, in vivo) | -0.472 |
| 9 | HGIIIFLKNVNVFPI  (HPV45-9) | 15 | HLA-DRB1*15:01, HLA-DRB1*07:01, HLA-DRB3*02:02, HLA-DRB3*01:01, HLA-DRB5*01:01, HLA-DRB4*01:01, HLA-DRB1*03:01 | 0.6794 | 0.5504 | No | No | Type 45: 100%  Type 18: 80% | 3.5 hours (mammalian reticulocytes, in vitro).  10 min (yeast, in vivo).  >10 hours (Escherichia coli, in vivo) | 1.313 |
| 10 | VGNPYFRVVPNGAGN  (HPV45-71) | 15 | HLA-DRB1*07:01, HLA-DRB5*01:01, HLA-DRB3*02:02, HLA-DRB1*15:01, HLA-DRB4*01:01, HLA-DRB3*01:01, HLA-DRB1*03:01 | 0.5344 | 0.1271 | No | No | Type 45: 100% | 100 hours (mammalian reticulocytes, in vitro).  >20 hours (yeast, in vivo).  >10 hours (Escherichia coli, in vivo) | -0.233 |
| 11 | GNPYFRVVPNGAGNK  (HPV45-72) | 15 | HLA-DRB5*01:01, HLA-DRB1*07:01, HLA-DRB3*02:02, HLA-DRB1*15:01, HLA-DRB4*01:01, HLA-DRB3*01:01, HLA-DRB1*03:01 | 0.5114 | 0.0296 | No | No | Type 45:100% | 30 hours (mammalian reticulocytes, in vitro).  >20 hours (yeast, in vivo).  >10 hours (Escherichia coli, in vivo) | -0.773 |
| 12 | HPFYNKLDDTESAHA  (HPV45-147) | 15 | HLA-DRB3*02:02, HLA-DRB5*01:01, HLA-DRB4*01:01,  HLA-DRB3*01:01, HLA-DRB1*07:01,  HLA-DRB1*03:01, HLA-DRB1*15:01 | 0.5102 | 0.1334 | No | No | Type 45: 100%  Type 18: 93.3%  Type 16: 73.3%  Type 59: 73.3% | 3.5 hours (mammalian reticulocytes, in vitro).  10 min (yeast, in vivo).  >10 hours (Escherichia coli, in vivo) | -1.233 |


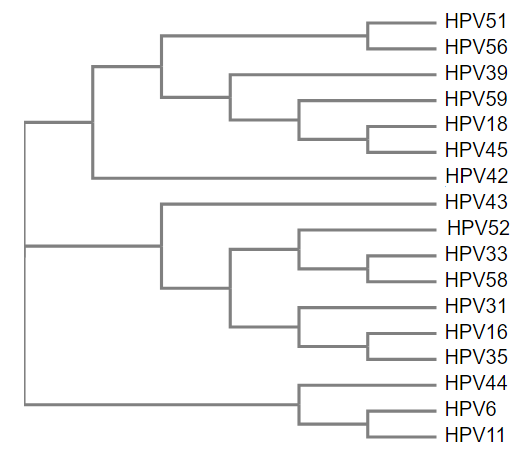


Figure S1. The guide tree of the Clustal Omega alignment of the five low-risk (HPV-6, 11, 42, 43, and 44) and 12 high-risk (HPV-16, 18, 31, 33, 35, 39, 45, 51, 52, 56, 58, and 59) human papillomaviruses.


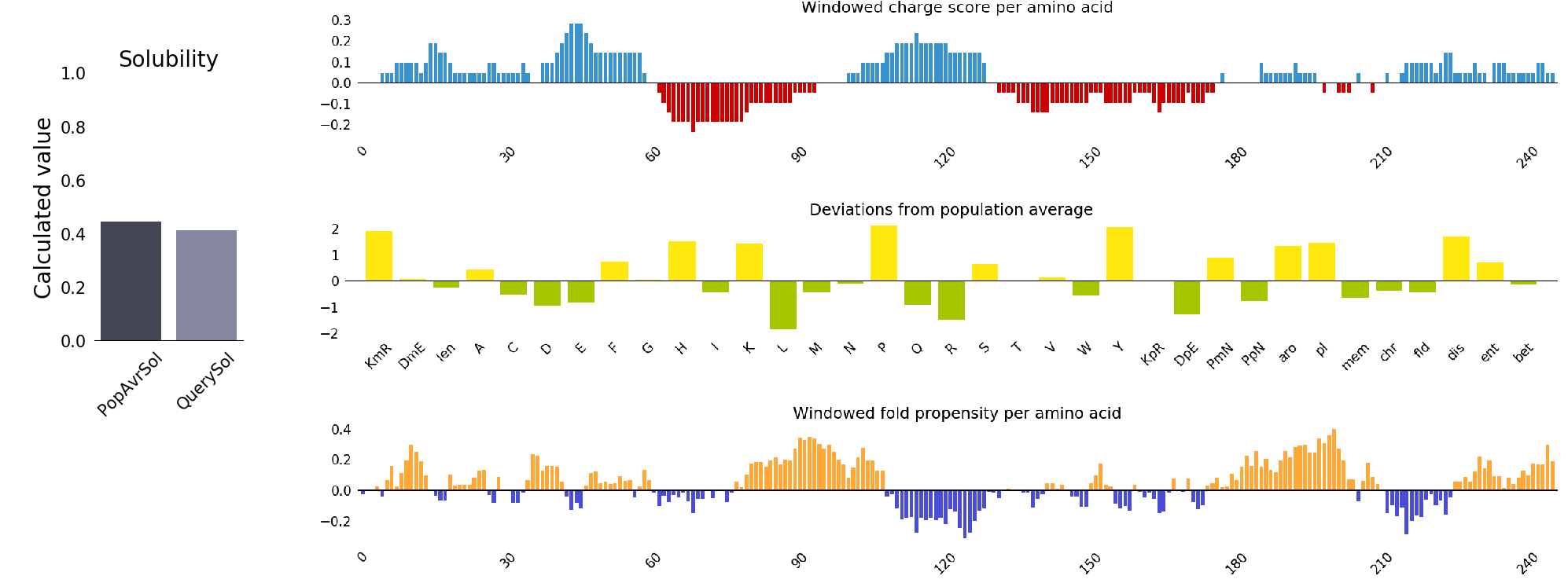


Figure S2. Predicted solubility of multiepitope vaccine candidate by Protein-Sol.


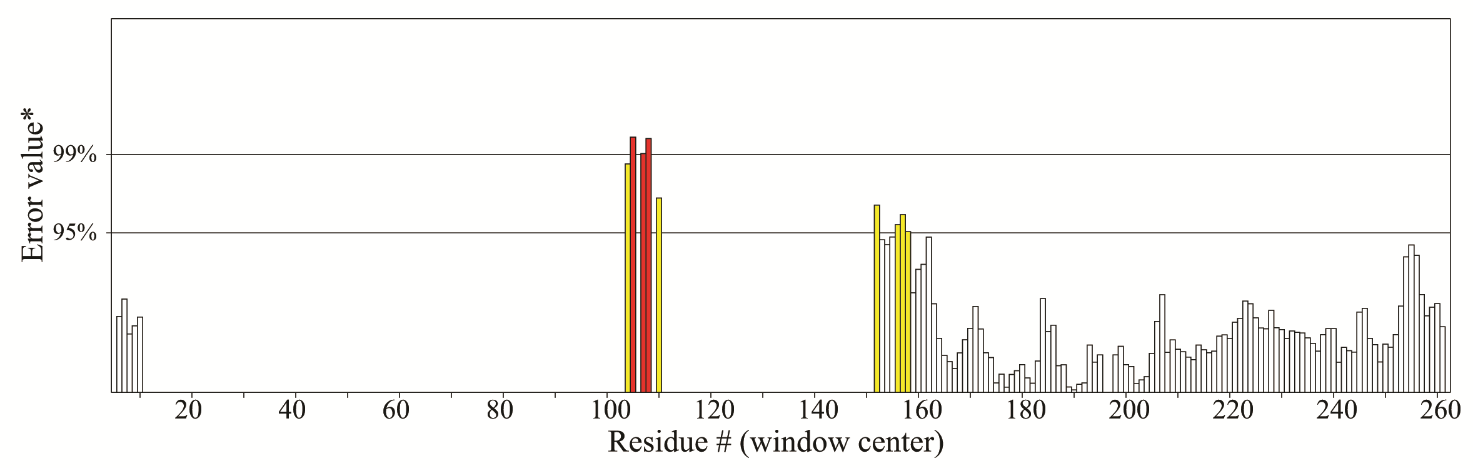


Figure S3. ERRAT result showing overall quality factor of the predicted structure of multiepitope vaccine candidate. Black bars illustrate the error region above 99%, gray bars denote the error region between 95% & 99% and white bars demonstrate the error region below 95%.


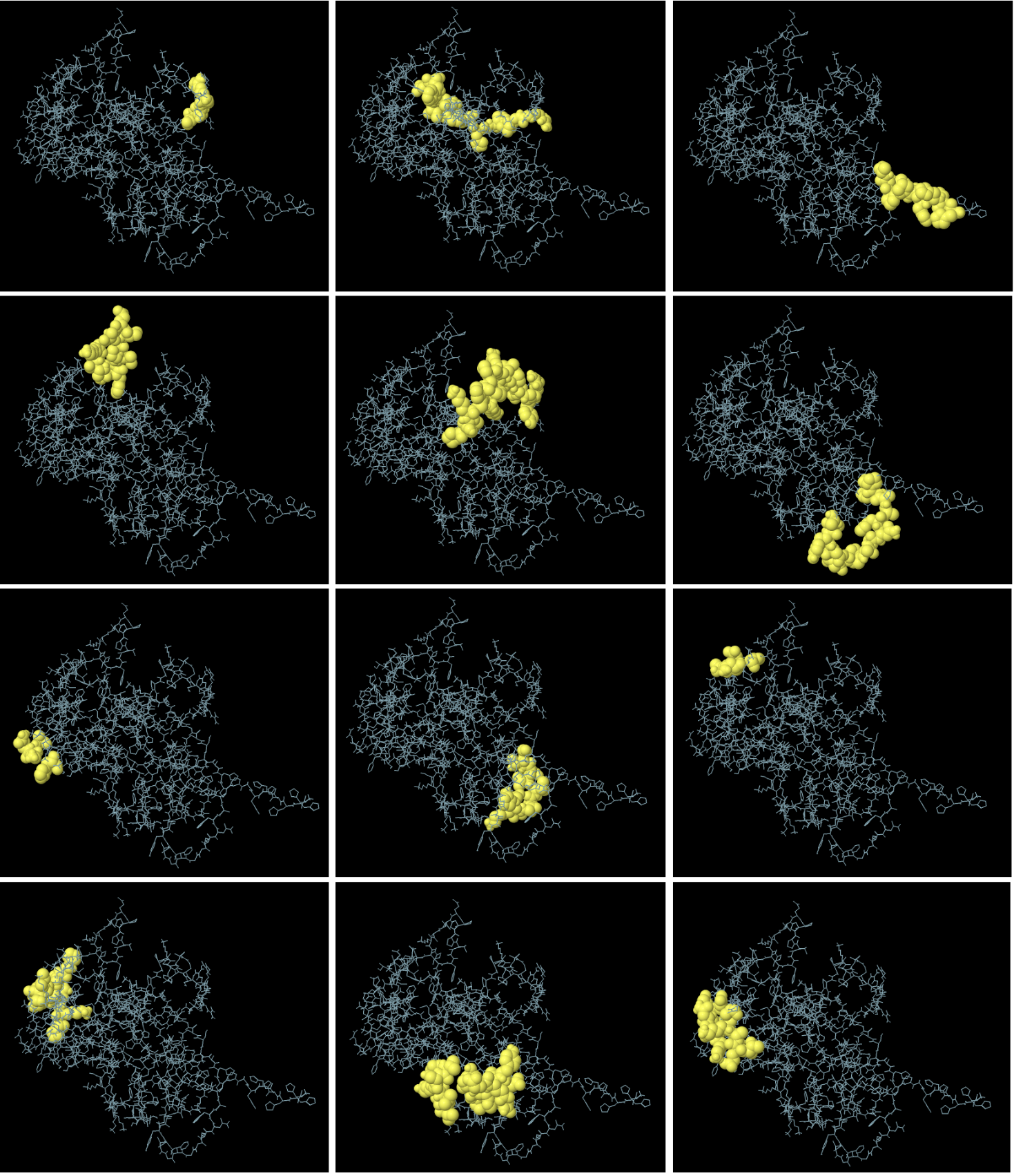


**Figure S4.** The graphical representation of discontinuous B-cell epitopes on the 3D model of the multi-epitope vaccine. The vaccine construct and discontinuous B-cell epitopes are depicted in gray sticks and yellow surfaces, respectively.
